# Inpatient cognitive analytic therapy for functional neurological disorder: A mixed methods four‐phase single‐case experimental design

**DOI:** 10.1111/papt.70002

**Published:** 2025-08-06

**Authors:** Stephen Kellett, Chris Gaskell, Isobel Dunning, Jordan Pope, Melanie Simmonds‐Buckley, Ben Lorimer

**Affiliations:** ^1^ Rotherham Doncaster and South Humber NHS Foundation Trust Doncaster UK; ^2^ Clinical and Applied Psychology Unit University of Sheffield Sheffield UK; ^3^ University of Exeter Exeter UK; ^4^ North Staffordshire Combined NHS Foundation Trust Trentham UK; ^5^ Clinical Psychology Training Programme University of East Anglia Norwich UK

**Keywords:** cognitive analytic therapy, FND, functional seizures, inpatients

## Abstract

**Objectives:**

To evaluate the effectiveness of inpatient cognitive analytic therapy (CAT) for functional neurological disorder and associated suicidality.

**Design:**

A single case experimental A‐B‐C plus follow‐up design. Intervention phases (A‐B‐C) were conducted on a psychiatric ward, with the 1‐month follow‐up (FU) phase in the community.

**Methods:**

The intervention was eight‐session CAT. The ABC phases of the SCED were synchronized with the reformulation, recognition and revision phases of the CAT. Idiographic anxiety, depression, burden, emotion regulation, distrust and frequency counts of epileptic and functional seizures were measured daily for *N* = 61 days across all the phases of the study. Recognition and revision of target problems (TPs) and target problem procedures (TPPs) were rated at each session. The PHQ‐9 and GAD‐7 were completed at assessment, end of therapy and follow‐up. A change interview was conducted at the point of discharge from the ward and at follow‐up.

**Results:**

The number of seizure‐free days did not change markedly. Recognition and revision of TP and TPPs increased positively over time and according to the phase of CAT. Daily depression significantly improved, but there was less change in other idiographic measures. There was a reliable improvement in PHQ‐9 and GAD‐7 scores. Personal changes at end of therapy and at follow‐up were described in the change interview as important, impactful and unlikely without therapy.

**Conclusions:**

The outcome picture was complex, with evidence of significant change on some indices and little change on others. This suggests a partially effective intervention. Inpatient CAT and also CAT as an FND intervention both seem to hold promise, but clearly more controlled research is needed.

## INTRODUCTION

Functional neurological disorder (FND) is an umbrella term describing a collection of neurological problems/symptoms, including changes to motor control, awareness and/or perception, but occurring in the absence of any demonstrable nervous system structural abnormalities (Perez et al., 2021). This has been likened to a software versus hardware problem (Bègue et al., 2019). This metaphor means whereas epilepsy would be a problem with organic brain functioning, FND occurs in the presence of abnormal neurobiological functioning, but crucially also in the context of an ‘intact macroscopic brain structure’ (Bègue et al., 2019). FND is considered to represent the expression of involuntary responses to internal or external stressors in the context of ongoing problems with emotion regulation (Brown & Reuber, 2016; American Psychiatric Association, 2022). Therefore, limited extant skills in the processing and management of emotions has been proposed as the key moderator between historical psychosocial risk factors and the core features of FND (Pick et al., 2019). Common FND presentations include functional/dissociative seizures, functional movement problems, functional speech difficulties and functional cognitive disorder.

Functional/dissociative seizures (FDS) been described as paroxysmal episodes which superficially resemble epileptic seizures or syncope, but which are not associated with the same physiological changes and processes that define and underpin epilepsy (Reuber, 2008). There is some uncertainty regarding the true prevalence of FDS and FND due to methodological and measurement issues, but estimates suggest that FND is prevalent in approximately 50 persons per 100,000 (Carson & Lehn, 2016). FDS represents 8–12% of first presentations to neurology (Angus‐Leppan, 2008), 11% of seizures presenting at emergency departments (Dickson et al., 2017) and 20%–30% of those referred to epilepsy centres with intractable seizures (Bompaire et al., 2021). FDS are therefore a clinical problem which occur on the borders between neurology, psychiatry and clinical psychology (Bègue et al., 2019).

Whilst FND is diagnosed by neurologists, the three treatment options then offered typically include (in isolation or combination); psychological interventions, treatments delivered by allied health professionals (e.g. physiotherapy and speech therapy) and pharmacological treatment of any underlying mental health disorder (BPS, 2024; NHS Inform, 2024). These interventions are typically delivered on an outpatient basis. As the evidence base for the treatment of FND develops, psychological interventions are becoming increasingly recognized as the front‐line treatment option (Gaskell et al., 2024; Goldstein et al., 2020; Gutkin et al., 2020). The predominant psychological therapies used are cognitive behavioural therapy (CBT) and psychodynamic therapy (PDT; see Gutkin et al., 2020 for a review). These treatments differ vastly in terms of how they formulate FND and also their mechanisms of intended therapeutic action (Gutkin et al., 2020). Broadly, CBT uses a here‐and‐now approach to challenge core beliefs, broaden coping and alter any underlying maladaptive behaviours, whilst PDT uses a past‐present approach to generate insight into unconscious factors and expand the emotional bandwidth (Gutkin et al., 2020). When an inpatient treatment is deemed necessary, this takes place at specialist neurorehabilitation units, with the average length of admission being almost 20 weeks and the intervention then being biopsychosocial (Saunders et al., 2024).

Cognitive analytic therapy (CAT) is a brief, structured, transdiagnostic and integrative therapy, commonly used to treat complex and enduring mental health problems (Ryle & Kerr, 2003). CAT was originally conceived as an integration of cognitive theory (Kelly, 1955) and also object relations theory (Ogden, 1983) and was designed for use in public services. There is now meta‐analytic evidence of the effectiveness and acceptability of CAT for a range of mental health disorders (Hallam et al., 2021; Simmonds‐Buckley et al., 2022). CAT is time‐limited with 8, 16 or 24 weekly sessions (depending upon the patient/problem complexity), followed by a series of follow‐up sessions according to the length of intervention (Ryle et al., 2014). CAT is theoretically underpinned by a three‐phase approach of *reformulation*, *recognition* and *revision* which seeks to identify, monitor and then positvely change target problems (TPs) and associated target problem procedures (TPPs). In the context of FND, this would be developing an understanding of the developmental origins and current maintainers of the physical symptoms (i.e. reformulation), enabling better recognition of the roles and patterns maintaining the physical symptoms (i.e. recognition), and then working on active therapeutic changes (i.e. revision). This ‘past‐present’ focus of CAT is important as past maltreatment and past difficult life events are substantially more common in FND (Ludwig et al., 2018). During CAT, such factors would be reformulated in terms of how they continue to negatively influence self‐self, self‐other and other‐self relationships. The CAT competency model highlights how therapists can move through the three phases and also consistently manage ‘enactments’ in the therapeutic relationship (i.e. when the patient is relating to the therapist in ways that mirror previously important developmental relationships; Parry et al., 2021).

The evidence base for CAT in the treatment of adult FND comprises two uncontrolled case reports of outpatient therapy (Nasiri et al., 2015; Tinlin‐Dixon, 2024) and a small pre‐post case series. In Bearman's (2019) case series, CAT was delivered to *N* = 6 outpatients; two dropped out, but there were significant reductions on the Hospital Anxiety and Depression Scale, the CORE‐OM and the Work and Social Adjustment Scale for the completers. The design of all these studies lack sufficient internal validity and methodological credibility. Increased methodological rigour can be achieved through utilizing experimental small N methodologies, containing intensive idiographic and nomothetic measurement (i.e., conduct a single case experimental design, SCED; Hersen, 1990). Krasny‐Pacini and Evans (2018) described SCED as a range of experimental designs that seek to evaluate the effectiveness of an intervention. Machalicek et al. (2024) therefore defined the characteristics of SCEDs as being experimental studies that focus on the response of the individual patient to an intervention that seeks to scientifically evaluate effectiveness through designing causal inference time‐series studies. The core design features of SCED therefore preemptively seek to address threats to internal validity and so have clinical credibility and external validity (Yang et al., 2023). SCEDs evaluate effectiveness through utilizing a small number of patients, generating repeated and intensively measured idiographic and nomothetic outcomes, the sequential (or randomized) introduction of phases of an intervention, and also applying method‐specific statistical ‘non‐overlap’ analysis. Whilst SCED is not mentioned as an evaluation method in the Medical Research Council's framework for developing and evaluating complex interventions (Skivington et al., 2021), it would fit as an early‐stage evaluation method.

This current study created a mixed‐methods SCED by utilizing daily measured idiographic outcomes, sessional tracking of TPs/TPPs, nomothetic outcome measures at pre, post and follow‐up, and conducting two qualitative interviews. The design of the current SCED method was also mapped onto the three phases of CAT (i.e. creating an A/B/C design). SCED is the method of choice where the evidence base for intervention is in the early stages of development, as is the case of CAT for FND. The study also fits with the Pick et al. (2019) call for more controlled FND outcome research. The study was conducted on an inpatient psychiatric unit. This is therefore the first controlled evaluation of inpatient CAT (see Cavieres & Tan, 2024 for a review). The five study hypothesises were that CAT would: (1) significantly reduce the daily intensity of anxiety, depression, burden, emotion regulation problems and distrust, with these reductions being maintained at follow‐up; (2) significantly reduce the daily frequency of functional seizures, with seizure reduction frequency being maintained throughout follow‐up; (3) increase the recognition and revision of TPs/TPPs according to the phase of therapy; (4) enable a reliable and clinically significant improvement in post‐treatment depression and anxiety on nomothetic outcome measures that would be maintained over the follow‐up period; and (5) that change would be attributed to CAT.

## METHOD

### Ethics and design

The study is based on the single‐case reporting guidelines (SCRIBE; Tate et al., 2016). The participant was given an information sheet, provided written consent for the study to be conducted and reported, and ethical approval for the study was also granted (ref: 041077). The SCED design had four phases (A‐B‐C‐FU) with a 4‐week follow‐up phase, and the phases were reformulation (A), recognition (B), revision (C) and follow‐up (FU). The patient was not ‘selected’ for the study, but the measurement of nomothetic and idiographic outcomes were a part of routine evaluation practice. It is normal practice in SCED for consent to be sought after the intervention has been delivered (Råbu & Binder, 2025), because the measures are part of routine practice, and seeking consent prior to the intervention might bias the patient towards positive responding (e.g. in the “people pleasing” patient). The idiographic measures and the TPs and TPPs were collaboratively designed between the therapist and the patient in the first of the eight sessions.

### Idiographic outcome measures and analysis strategy

Six idiographic measures were collected daily throughout all phases of the study. The reformulation phase (A) lasted 13 days and contained two sessions; the recognition phase (B) lasted for 8 days and contained three sessions; the revision phase (C) lasted for 16 days and contained three sessions; and the follow‐up phase (FU) lasted 24 days. Phases A, B and C were delivered on a psychiatric inpatient ward, and follow‐up was conducted after discharge into the community from the ward. Therefore, the study had a time series of *N* = 61 days. In terms of data completeness, there was no missing data from the A or B phases; the C phase had 2 days missing and the FU phase had 9 days missing. The six idiographic outcome measures were all measured on Likert scales: feeling anxious today (0 not at all to 100 unbearably anxious), feeling depressed today (0 not at all to 100 suicidal), how I have been with my emotions (0 cut‐off and hiding to 100 overwhelmed), feeling ‘sensitive’ today around people (0 trusting to 100 untrusting) and feeling like a burden today (0 not at all to 100 totally). Epileptic and functional/dissociative seizures were measured with daily seizure counts. Interrupted time‐series plots were used to illustrate the shape of change in idiographic outcomes over the phases of the study. Effectiveness of change in idiographic outcomes was assessed using three non‐overlap statistics: the percentage of data points exceeding the median (PEM; Ma, 2006), the percentage of all non‐overlapping data (PAND; Parker et al., 2007) and non‐overlap of all pairs (NAP; Parker & Vannest, 2009). Non‐overlap outcomes were interpreted using Scruggs and Mastropieri's (1998) guidelines: <70% = questionable/ineffective treatment, 70%–90% = moderately effective treatment and >90% = highly effective treatment.

### Target problems and target problem procedures

Three TPs and associated TPPs (one snag and two dilemmas) were rated at each session for recognition (0—‘cannot see the pattern’ to 100—‘spotting it really well’) and revision (0—‘have not been able to change it’ to 100—‘changing it very effectively’). The snag was ‘*I want to move on with my life, but I am clinging to my Grandad*.’ The emotion dilemma was ‘*I am either completely cut off from my feelings or I am consumed by them*’ and the abandonment dilemma was ‘*I either look after other people or I abandon myself*.’

### Nomothetic outcome measures and associated analysis strategy

Two nomothetic outcome measures were completed prior to session 1, after session 8 and at follow‐up. Change on these measures was assessed using reliable and clinically significant change criteria (RCSC; Jacobson & Truax, 1991).

### Patient Health Questionnaire‐9

The PHQ‐9 is a 9‐item (0–3) scale, based on the nine DSM‐V criteria listed under criterion A for major depressive disorder (Kroenke & Spitzer, 2002). The PHQ‐9 is a widely used measure for case identification due to its reliability and validity. Caseness for depression is a score of ≥10. PHQ‐9 scores are interpreted as minimal depressive symptoms (1–9), mild depressive symptoms (10–14), moderate depressive symptoms (15–19) and severe depressive symptoms (20–27). For reliable change, scores need to be reduced by 6+ points, and for clinically significant change, the post score needed to be <10.

### General Anxiety Disorder‐7

The GAD‐7 is a 7‐item (0–3) scale that is used to measure the severity of generalized anxiety disorder (Spitzer et al., 2006). The overall score can range from 0 to 21, with cut‐off scores for mild, moderate and severe anxiety symptoms being 5, 10 and 15, respectively. Anxiety caseness is a score ≥8. The GAD‐7 is valid and reliable, having been validated within primary care, general populations and adolescents (Mossman et al., 2017). For reliable change, scores need to be reduced by 4+ points, and for clinically significant change, the post score needed to be <8.

### Change interview

This semi‐structured interview (Rodgers & Elliott, 2015) was conducted by a Physician Associate (i.e. to reduce the bias of the therapist conducting the interview) on the hospital site at the end of treatment and at follow‐up. Each interview lasted for 30 minutes and was supported by the TP/TPP graphs and idiographic outcome graphs. The Change Interview identifies whether change has occurred during therapy, what those changes were and whether they were connected (or not) to the therapy delivered (i.e. the interview sceptically tries to work against the expectation that therapy has been helpful; Thompson & Harper, 2012). The patient participant was invited to review the CAT intervention, identify associated changes and rate each change in terms of expectation (i.e. 1 expected to 5 surprising), possibility (i.e. 1 unlikely to have changed without therapy to 5 likely to have changed without therapy) and personal importance (1 not at all to 5 extremely).

### The patient

The participant was a 33‐year‐old female, admitted to a mixed‐sex 18‐bedded adult inpatient psychiatric ward following an aborted suicide attempt and concerns about the risk of imminent and further self‐harm. The admission was not to treat the FND necessarily, but certainly to manage the risk to self. The participant felt that they were not coping with their life due to the FND, felt trapped and hopeless and therefore wanted to end their life. At admission, the participant had recently moved to the region, was currently unemployed, but had recently been in long‐term employment in a hospitality role. No previous psychiatric admissions. The weekly purposeful admission meeting on the ward identified the need for psychological input. The participant received a diagnosis from the inpatient Consultant Psychiatrist of co‐morbid anxiety and depression (with suicidal ideation) and complex post‐traumatic stress disorder (cPTSD).

The participant was born into a family comprised of a mother, father and sibling. There was a strong family history of epilepsy on both paternal and maternal sides. Due to the participant's parents struggling with alcoholism (with domestic violence), the participant was moved away to live with their grandparents from a young age, with a sibling staying with the parents. This created both a sense of abandonment and also guilt concerning the sibling, early emotional repression, and an over‐focus on meeting the needs of others. The participant had a close bond with their grandparents (reformulated as a perfect care bubble), and particularly her grandfather. The participant cared for their grandfather in the final months of his life. When their grandfather died, this left a huge sense of loss, grief and pain, which was exacerbated also by the loss of a child. The participant was in a long‐term heterosexual relationship that was supportive, and the couple had one child. The participant felt untrusting of their partner and was paranoid that the partner might leave at any moment. The participant had struggled with anxiety and depression for many years, but more severe anxiety and depression coincided with FND onset. The FND also disrupted the ability of the participant to care for their daughter and also trust in herself that she could do this safely.

Throughout the admission, the participant was prescribed medications for mental health and epilepsy. The participant was prescribed Propranolol 120 mg and Amitriptyline 20 mg to aid sleep. For depression, the participant was switched from Mirtazapine 45 mg to Citalopram 30 mg between sessions 7 and 8 of CAT. The epilepsy was treated with Clobazam 10 mg, Lamotrigine 400 mg, and Levetiracetam 2500 mg. In terms of previous psychological interventions, the participant had engaged in person‐centred experiential counselling for depression. They attended three sessions before they were discharged from that service due to being accepted onto a specialist psychotherapy for FND waiting list attached to a local neurology department. There had been no response to the counselling.

In terms of neurological symptoms, the first tonic–clonic seizure occurred in August 2022. The first seizure had a prodromal phase (‘feeling funny’), an ictal phase including jerking movements in both arms and legs, incontinence and tongue‐biting followed by a post‐ictal phase. An epilepsy diagnosis was made by a Neurologist in 2022. Vacant seizures were reported in February 2023 and a mixture of ‘convulsive’ seizures and vacant episodes (at least daily) at the time. MRI of the head was found to be normal in 2023. Videos from professionals while the participant was admitted to an adult medical unit in 2023 suggested non‐epileptic seizures. Neurologists therefore suggested considering at that time a new diagnosis of epileptic and functional seizures. Levetiracetam loading was required during this admission. An EEG in May 2023 was conducted whilst on 350 mg Lamotrigine OD and Clobazam 10 mg. Three vacant episodes occurred during the EEG, but no epileptiform activity was recorded. Another EEG performed in October 2023 did not capture any clinical events, but did capture some frequent bursts of slow‐wave activity over both temporal regions, with a right‐sided emphasis. Occasionally, the slow waves had a sharpened configuration. It was commented that these were not specific abnormalities, but could suggest temporal lobe epilepsy. In November 2023, the dual diagnosis of epileptic and functional seizures was considered the main conclusion of a clinical review. A diagnosis of focal epilepsy with focal to bilateral tonic–clonic seizures and non‐epileptic attack disorder (FND) with vacant spells and collapsing with asynchronous whole‐body jerking was then finalized in December 2023 by a Consultant Neurologist.

The epileptic seizures contained the following symptoms: generalized tonic–clonic, facial tension, duration 2–3 min, changes to oxygen saturations, blood pressure and tachycardia ~ described cyanosis, confusion, combativeness and unrousable postictal with immovable rigidity. The functional seizures contained the following symptoms: full body shaking, minimal limb rigidity, able to open eyes and be moved, some awareness of current conversations, able to respond at times, often occurring following an epileptic seizure, more prolonged in duration (i.e. 5–6 min), minimal change to physical observations, with tachycardia being the only change, minimal confusion postictal, and able to talk and self‐care almost immediately post a functional seizure.

### Treatment

Treatment was delivered on an mixed‐bedded adult inpatient psychiatric ward provided by the National Health Service in the United Kingdom. The therapist was a male Consultant Clinical Psychologist and CAT psychotherapist, supervisor and trainer. There was monthly clinical supervision for the intervention from a CAT practitioner. Following a screening session, the patient was allocated to the ‘Sheffield model’ of 8‐session CAT (Kellett et al., 2024). All sessions were biweekly on the ward, were 50 min in length and all eight sessions were attended. The treatment protocol was the session‐by‐session guideline designed for the RELATE feasibility randomized trial of CAT for self‐harm (Taylor et al., 2024), that has been adapted for inpatient delivery (Kellett, 2024).

As per the eight‐session version of CAT, the first two sessions were focal to assessment (e.g. taking a history); they did not contain any treatment elements, and the narrative reformulation (NR) was shared at session 3. The NR identified links between past and present, highlighting possible enactments and named the TPs and TPPs (Ryle & Kellett, 2018). To remain consistent with previous CAT SCED research (Kellett et al., 2022), the NR signalled the end of reformulation and the start of recognition (i.e. shifting from the A to B phase in the SCED).

As per treatment guidelines, the main components of CAT (i.e. NR, sequential diagrammatic reformulation [SDR] and goodbye letters exchanged between patient and therapist at termination) were present (Ryle & Kerr, 2003). Each component was reviewed at clinical supervision. Online supporting information report the SDR and the change map. A change map is constructed during the revision stage and contains healthy recrprocal roles and procedures. The most common enactment was the ‘perfect care’ enactment and analysing how this interacted with the functional seizures. The patient was discharged from the ward with a therapy pack containing the NR, SDR, TP/TPP graphs, idiographic time series graphs, nomothetic graphs, the change map, and goodbye letters. The follow‐up ratings on nomothetic measures and the idiographic measures were collected at follow‐up and then added to the previous phases and emailed to the patient.

## RESULTS

### Idiographic outcomes

**FIGURE 1:**
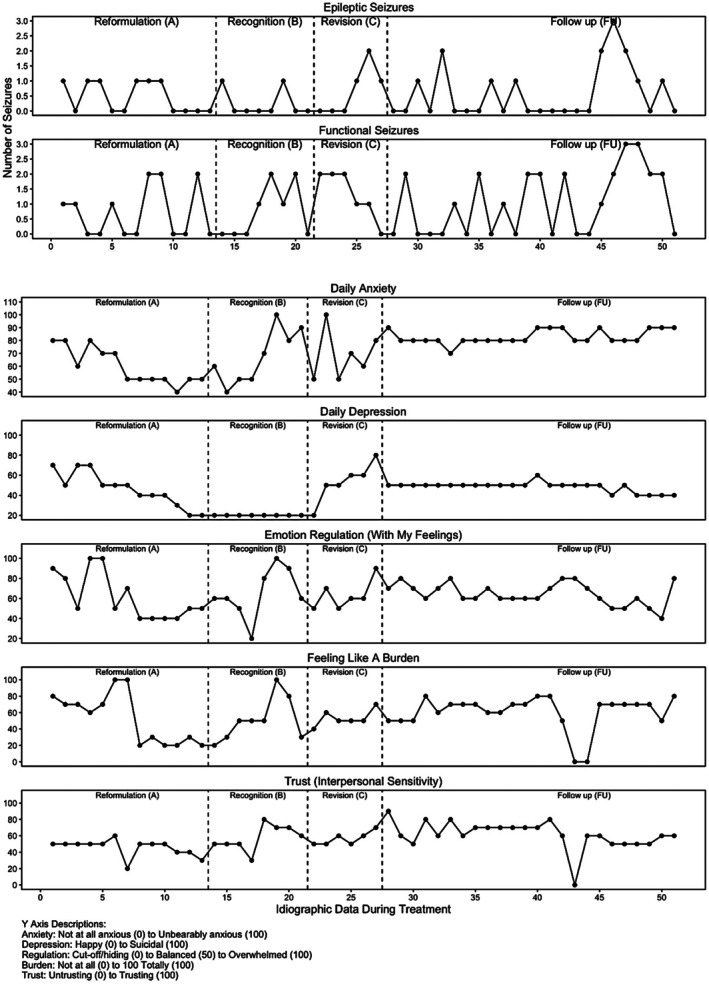
Time series plots of idiographic outcomes over the phases of the study.

Figure 1 presents time‐series plots for each idiographic outcome measure, and Table 1 provides a summary of descriptive statistics for each of the four phases of the study. Across these time‐series plots, there was no clear immediate change or notable shift in the level of daily intensity or frequency of idiographic outcome measures between any adjacent phase pairings. The occurrence rate of seizures (epileptic versus functional) per CAT phase was 6/9 (reformulation phase), 2/6 (recognition phase), 8/14 (revision phase) and 10/19 (follow‐up). Days free of epileptic seizures per length of phase were: 6/13 (46.15%) days‐free during reformulation, 6/8 (75.00%) days‐free during recognition, 10/16 (62.50%) days‐free during revision and 6/24 (25.00%) days‐free during follow‐up. The days‐free of functional seizures per length of phase were 7/13 (53.84%) days during reformulation, 4/8 (50.00%) days during recognition, 7/16 (43.75%) days during revision and 5/24 (20.83%) during follow‐up.

**TABLE 1:** Descriptive statistics for idiographic measures over the four phases.

	Reformulation Phase			Recognition Phase			Revision Phase			Follow up Phase		
	*N*	*Mean*	SD	*N*	*Mean*	SD	*N*	*Mean*	SD	*N*	*Mean*	SD
Epileptic seizures	13	0.46	0.52	8	0.25	0.46	6	0.67	0.82	24	0.58	0.88
Anxiety	13	60.00	14.14	8	67.50	21.21	6	68.33	19.41	24	82.92	5.50
Depressed	13	46.15	17.10	8	20.00	0.00	6	53.33	19.66	24	48.33	4.82
Feeling like a burden	13	53.08	30.93	8	51.25	26.96	6	53.33	10.33	24	60.42	21.16
Trust	13	45.38	10.50	8	57.50	15.81	6	56.67	8.16	24	61.67	17.11
Functional seizures	13	0.69	0.86	8	0.75	0.89	6	1.33	0.82	24	1.04	1.08
Emotion regulation	13	61.54	23.40	8	65.00	25.07	6	63.33	15.05	24	64.58	11.03

*Note*: *N* represents complete data. The revision phase has 2 data points missing and the follow up phase has 9 data points missing.

Table 2 summarizes the non‐overlap analysis of the idiographic outcomes. When the recognition phase was compared to the baseline reformulation phase, there was an immediate improvement in feeling less depressed (NAP = 92%, PEM = 100%, PAND = 75%). Each of the remaining idiographic measures had lower improvement rates, with at least one non‐overlap statistic falling below 60%. The emotion regulation idiographic measure showed a trend towards the mid‐point of the scale across the phases and so would be evidence of positive change. The deterioration on the time‐series graphs during the revision phase on the idiographic outcome measures appeared to be due to the participant struggling with the discharge process (i.e. due to a sudden change in medical consultant at that time) and experiencing this as an abandonment.

**TABLE 2:** Non‐overlap statistics for idiographic measures comparing phases of CAT (except sensitivity which did not suit an overlap analysis).

	Non overlap of all pairs (NAP)			PAND (%)	PEM (%)
	%	SE	95% CI		
Reformulation vs. Recognition					
Epileptic seizures	61	0.12	(36%–80%)	62	38
Anxiety	40	0.14	(20%–65%)	62	25
Depressed	92	0.06	(68%–98%)	90	100
Feeling like a burden	50	0.13	(27%–72%)	62	75
Functional seizures	48	0.13	(26%–71%)	62	25
Emotion regulation	26	0.12	(11%–52%)	62	31
Reformulation vs. Revision					
Epileptic seizures	44	0.15	(21%–70%)	68	25
Anxiety	37	0.14	(17%–64%)	68	17
Depressed	36	0.15	(16%–63%)	68	33
Feeling like a burden	50	0.14	(26%–74%)	68	75
Functional seizures	29	0.13	(12%–58%)	68	08
Emotion regulation	21	0.10	(7%–49%)	68	25
Reformulation vs. Follow up					
Epileptic seizures	50	0.09	(32%–69%)	65	31
Anxiety	08	0.05	(3%–25%)	65	00
Depressed	43	0.12	(26%–62%)	73	58
Feeling like a burden	43	0.11	(26%–62%)	70	35
Functional seizures	42	0.09	(25%–61%)	65	23
Emotion regulation	13	0.06	(5%–32%)	65	15
Recognition vs. Follow up					
Epileptic seizures	42	0.09	(23%–64%)	75	31
Anxiety	28	0.15	(13%–51%)	78	00
Depressed	00	0.00	(0%–00%)	75	00
Feeling like a burden	34	0.14	(17%–57%)	78	19
Functional seizures	43	0.11	(24%–65%)	75	46
Emotion regulation	40	0.13	(22%–63%)	75	25

Abbreviations: PAND, percentage of all non‐overlapping data; PEM, percentage of data exceeding the median; SE, standard error.

### Target problem and target problem procedures outcomes

**FIGURE 2:**
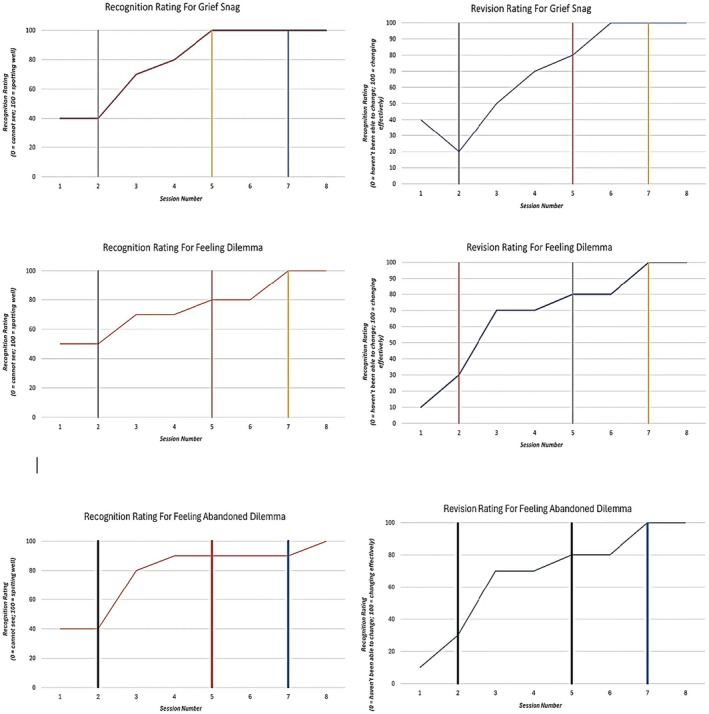
TP and TPP sessional ratings for reformulation (sessions 1–2), recognition (sessions 3–5) and revision (sessions 6–8).

Figure 2 contains the TP/TPP recognition and revision ratings. All three TP/TPPs show the same pattern of increasing recognition and revision following flat baselines during the reformulation phase. The mean ratings for recognition and revision of the grief snag per phase were reformulation phase (40% and 30%), recognition phase (83.30% and 66.60%) and revision phase (100% and 100%). The mean ratings for recognition and revision of the emotion dilemma were reformulation phase (50% and 20%), recognition phase (73.30% and 73.30%) and revision phase (93.30% and 93.30%). The mean ratings for recognition and revision of the abandonment dilemma were reformulation phase (40% and 20%), recognition phase (86.66% and 73.33%) and revision phase (93.33% and 93.33%).

### Nomothetic outcomes

The GAD‐7 was 20 at intake, 12 at the end of CAT and 10 at follow‐up. The PHQ‐9 was 26 at intake, 16 at the end of CAT and 14 at follow‐up. This would represent a reliable (but not clinically significant) reduction in anxiety that was maintained at follow‐up and a reliable (but not clinically significant) reduction in depression that was also maintained at follow‐up.

### Change interview

Three main changes were reported at discharge (i.e. expressing feelings, 'good enough' stance and improved self‐confidence) and two main changes were reported at follow‐up (i.e. ‘feeling feelings’ and increased honesty with others). These changes are reported in Table 3. The changes were rated as unexpected, connected to the therapy and personally meaningful.

**TABLE 3:** Changes reported and rated at the end of CAT and again at follow‐up.

	Change was: 1 expected, 3 neither expected nor unexpected, 5 surprised by the change	Change without therapy: 1 very unlikely, 3 neither, 5 very likely	Personal importance: 1 not at all, 2 slightly, 3 moderately, 4 very, 5 extremely
Reported at the end of CAT			
Able to my effectively express feelings	5	1	5
Having a ‘good enough’ is enough stance	3	1	5
Self‐confidence improved	5	1	5
Reported during follow‐up			
Communication with others—able to be more honest	4	1	4
Permission to ‘feel’ my feelings	4	1	4

## DISCUSSION

This study has evaluated the clinical effectiveness and durability of inpatient CAT for FND and associated suicidality using a mixed‐methods A‐B‐C‐FU SCED. There have been no previous controlled evaluations of the effectiveness of inpatient CAT (Cavieres & Tan, 2024) nor FND (Bearman & Jenaway, 2024) and so the study represents a useful early step in early evidence generation. The SCED design used here has greater internal validity than the bi‐phasic (i.e. quasi‐experimental) A‐B designs that are more commonly used (Kazdin, 1978). This is because change during both recognition and revision phases compared to reformulation tested whether active CAT treatment affected change, thus reducing the risk of attributing change to other factors (e.g. passage of time and regression to the mean; Rizvi & Nock, 2008). The mixed‐methods evaluation produced a network of idiographic measurement (TP/TTP sessional measures and daily measures of mood/wellbeing and seizure frequency), nomothetic measurement and qualitative interviewing. When results were synthesized across this network, this produced a nuanced evaluation of change. The selection of outcome measures being a blend of FND core symptoms and associated psychological distress fits with best practice in outcome measurement for FND (Pick et al., 2020).

Baselines on idiographic measures were generally stable, but there was less evidence of subsequent change, whereas the TP/TPP ratings of recognition and revision showed change, and this was synchronized with the phase of CAT. The changes created by the CAT were personally meaningful, unexpected and crucially, would not have occurred without the therapy. The difference between TP/TPP ratings (evidence of change) and the idiographic measures (less evidence of change) was presumably due to the TP/TPPs being the focus of the work, as is consistent with the CAT competency framework (Parry et al., 2021). The rate of functional seizures did not reduce due to CAT and the patient was therefore not seizure‐free at the end of treatment or follow‐up. Sixty per cent of patients are not seizure‐free at the end of psychological therapy (Gaskell et al., 2024). The reliable reduction due to treatment on the nomothetic anxiety and depression measures would fit with the evidence base that psychological therapy for FND can reduce associated distress (Gaskell et al., 2023). It is worth noting that where change was apparent (i.e. TP/TTPs, nomothetic measures and interview), then this was still achieved in the briefest version of the CAT model. All eight sessions were attended; the median number of inpatient CAT sessions is four (Cavieres & Tan, 2024) and there is meta‐analytic evidence of the differential acceptability of CAT (Simmonds‐Buckley et al., 2022).

It is worth noting that despite there being only one active treatment phase during the study (i.e. the ‘C’ revision phase of CAT), it was still the same therapy (i.e. CAT) that was being delivered, with the same NR and SDR. It was evident from the time‐series graphs that the patient participant found the discharge process from the ward difficult, and this affected idiographic outcomes also during the follow‐up phase. Consistent efforts were made to prepare the participant for the termination of CAT (there was no impact of ending on the TP/TPP ratings), but the wider sense of abandonment from the ward at the time influenced the idiographic outcomes. The overall outcome would be that of a partially effective psychological intervention. This conclusion regarding partial effectiveness is based on evidence of lack of insulation to the difficulties that occurred at the point of discharge and during the follow‐up, and lack of marked change in the rate of functional seizures. The recording of a partially effective outcome still contributes to the small evidence base for CAT for FND and in a study with more internal validity than has been previously achieved (Bearman, 2019; Nasiri et al., 2015; Tinlin‐Dixon, 2024). What CAT appeared to provide in terms of change were the skills for the patient to approach, process and so contain previously unmanageable feelings, and also share with others what they were feeling, and in doing so communicate more effectively what it was that they wanted or needed. The CAT tools were passed (with permission) onto the care coordinator in the community team on discharge (via a joint meeting with the patient) so that the community team could then continue to support the patient in a manner that was informed by the SDR and the change map. The recording of a partially effective outcome is also because the GAD‐7 and PHQ‐9 did not meet criteria for clinically significant change. The suicidality that created the decision to admit the patient was absent at the point of discharge and follow‐up.

The close alignment of the patient and the therapist on the collaborative design of the idiographic measures (Kellett & Beail, 1997) meant that completion of daily idiographic measures was not particularly burdensome, and this appears the case even in an inpatient psychiatric ward context. The patient found the graphing of TP/TPPs particularly useful and reinforcing. Sessional tracking of TP/TPPs enables ameliorative actions being taken when the patient is ‘off‐track’ in relation to recognition and revision (Schilling et al., 2021). Because exploration of enactments is a core CAT competency (Parry et al., 2021) then when a patient is ‘off‐track’ in terms of TP/TPP progress, then this can also be explored as a potential enactment. In terms of external validity, Murad et al. (2018) would categorize this SCED as having low generalizability (i.e. due to concerns regarding applying the results to other FND in or outpatients), whilst having high applicability (i.e. being able to draw useful inferences regarding providing integrative psychotherapy to FND patients). Whilst the nomothetic measures and the ABC with follow‐up phases of the study could be easily replicated, SCED is, by definition, dependent on the creation of face‐valid idiographic measures. Hence, what made sense to create, measure and monitor idiographically and intensively during the current study, would have poor face validity with other FND patients. However, recent methodological advances mean that single case meta‐analysis is now possible, and this retains the idiographic focus and appeal of the method (Moeyaert et al., 2023).

### Limitations and future methodological directions

All the outcome data was self‐report which limits confidence in the reliability of the outcomes reported due to the potential for social desirability bias (Arnold & Feldman, 1981). Having at least one TP and associated TPP that more directly reflected the FND would have been useful. The wording of the dilemma ‘either I care for others or abandon myself’ could have been improved. For example, emphasizing more the negative aspects at either end of the dilemma (e.g. “either I care totally for others and completely ignore myself or I care totally for myself, feel selfish and ignore others”). Co‐production of the research would have been useful (Needham, 2008). The introduction of a withdrawal phase would have strengthened the design (see Kellett et al., 2022 for an example of a withdrawal SCED design using CAT). The 16 or 24‐session version of the CAT model might have been more effective and so future research should compare outcomes for FND with differing CAT treatment lengths. The length of typical psychiatric admissions makes the 16 and 24 session approaches less viable. Supplementary informant data would have increased the internal validity of the method, such as the ward staff rating the patient in terms of mood and risk, or the partner rating openness (see Kellett & Totterdell, 2013 for an example SCED using partner ratings as idiographic outcomes). A wider selection of nomothetic outcome measures would have been useful. For example, the Scale for Suicide Ideation (e.g. the SSI; Beck et al., 1979) to more closely capture changes to dynamic risk issues. The follow‐up period was relatively brief and future studies need to have longer follow‐up periods. Sampling clinical competency would have been useful via the Competency in CAT measure (Bennett & Parry, 2004) and future studies should achive this. The study methodology could have been improved through the addition of an idiographic control measure and also a generalization nomothetic measure (Krasny‐Pacini & Evans, 2018). Future SCEDs with FND patients may seek to utilize cross‐over designs where there could be random allocation to CAT versus CBT or psychodynamic phases for example (Jones & Kenward, 2014). Rigorously designed SCEDs do evaluate effectiveness, but repeated replication across researchers, participants and contexts is still needed (Machalicek et al., 2024).

## CONCLUSIONS

This innovative and methodologically unique study has indexed a partially effective outcome of CAT for a patient with FND conducted during an inpatient psychiatric admission due to associated suicidality. The evidence base for inpatient CAT needs to be developed (Cavieres & Tan, 2024) as currently, despite CAT being delivered to inpatients and used as an organizational intervention with clinical teams, little is known about its clinical effectiveness. The study was a rigorous mixed‐methods SCED, and this heightens confidence in the conclusion drawn that CAT was partially effective. This research also supports the continued evaluation of CAT for FND (Bearman & Jenaway, 2024). SCED will be a valuable tool in these clinical endeavours before moving on to more group‐based evaluation designs.

## AUTHOR CONTRIBUTIONS

**Stephen Kellett:** Conceptualization; methodology; data curation; writing – original draft; writing – review and editing; formal analysis. **Chris Gaskell:** Writing – original draft; writing – review and editing; formal analysis; data curation. **Isobel Dunning:** Project administration; data curation; formal analysis. **Jordan Pope:** Project administration. **Melanie Simmonds‐Buckley:** Formal analysis; writing – review and editing; writing – original draft; methodology. **Ben Lorimer:** Writing – original draft; writing – review and editing.

## FUNDING INFORMATION

None.

## CONFLICT OF INTEREST STATEMENT

None.

## Supporting information


Data S1.

## Data Availability

The data that support the findings of this study are available on request from the corresponding author. The data are not publicly available due to privacy or ethical restrictions.
