# Supplementary material for: Inpatient cognitive analytic therapy for functional neurological disorder: A mixed methods four‐phase single‐case experimental design
Source: Psychol Psychother. 2025 Aug 6;98(4):1033–49. doi: 10.1111/papt.70002 (PMC12617480; doi:10.1111/papt.70002)
Supplement: Supplementary file 1 — Data S1. [file PAPT-98-1033-s001.pptx]

## Slide 1
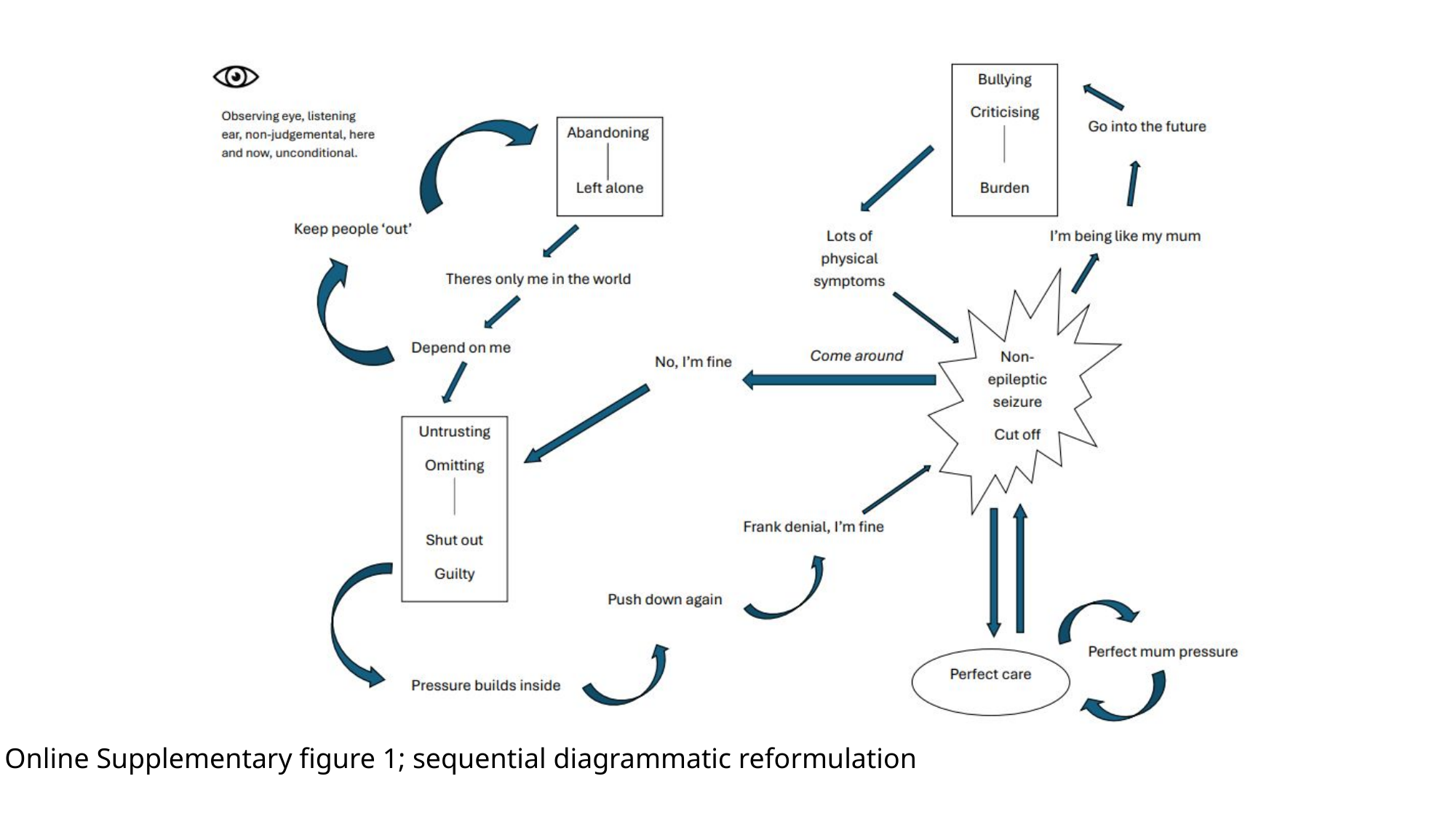

Online Supplementary figure 1; sequential diagrammatic reformulation

## Slide 2
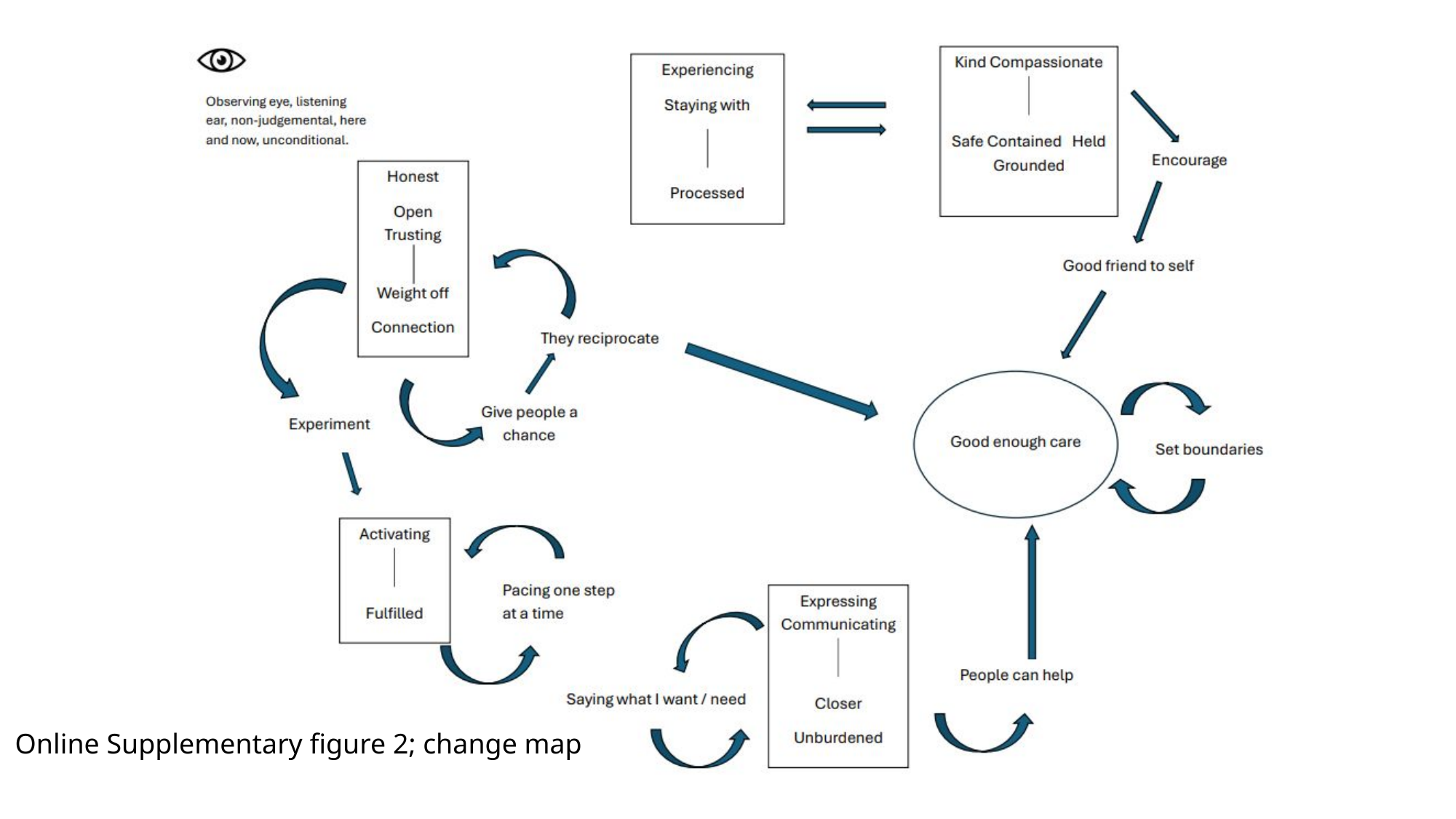

Online Supplementary figure 2; change map
